# Supplementary material for: Detecting and Treating Psychosocial and Lifestyle-Related Difficulties in Chronic Disease: Development and Treatment Protocol of the E-GOAL eHealth Care Pathway
Source: Int J Environ Res Public Health. 2021 Mar 23;18(6):3292. doi: 10.3390/ijerph18063292 (PMC8005221; doi:10.3390/ijerph18063292)
Supplement: Supplementary file 1 [file ijerph-18-03292-s001.zip › 20210322_ijerph-1139819_S1_proofreading.docx]

**Supplementary File 1**

**Screening questionnaire selection and development**

***Part 1: Questionnaires for Increased-Risk Profile Identification.*** First, to screen for psychological distress, we selected the nine-item Patient Health Questionnaire depression scale [PHQ-9; 1] and the seven-item Generalized Anxiety Disorder scale [GAD-7; 2]. The scales measure depressive and anxiety symptoms, such as “Little interest or pleasure in doing things” or “Feeling nervous, anxious or on edge”. Respondents are asked how much each symptom has bothered them over the past 2 weeks, with response options from 0 (“not at all”) to 3 (“nearly every day”). The PHQ-9 and GAD-7 have been used in numerous studies and incorporated into a variety of clinical practice guidelines for medical and mental health care settings [3]. Second, health-related quality of life (HRQoL) and fatigue are associated with poor health outcomes [4] and may indicate an increased-risk profile of experiencing psychosocial barriers for a healthy lifestyle. Therefore, we included the Shortened Fatigue Questionnaire [SFQ; 5] and the RAND 36-item Short Form Health Survey [RAND SF-36; 6] in the first screening part. The SFQ assesses fatigue in four items (e.g. “I feel tired”) with response options on a 7-point scale. The RAND SF-36 is a 36-item questionnaire assessing eight HRQoL dimensions. Physical HRQoL consists of the subscales physical functioning, role limitations due to physical health problems, pain, and general health perception. Mental HRQoL consists of the subscales vitality, social functioning, role limitations due to emotional health problems, and mental health.

Second, to determine which variables for lifestyle behaviors should be included, we consulted international guidelines for lifestyle-related diseases [7, 8]. Such guidelines are based on scientific evidence, among other things regarding the potential benefits of the healthy lifestyle behaviors for physical and psychological health outcomes (e.g. by slowing down disease progression, diminishing complication risks, or improving quality of life). Key lifestyle variables are physical activity, BMI, eating behaviors, smoking, and medication adherence. To measure physical activity, we selected the Short Questionnaire to Assess Health-enhancing physical activity [SQUASH; 9], in which respondents can indicate how many days per week, average minutes per day, and at which intensity they practice commuting activities, leisure time, household, and activities at work or school. To measure BMI, we included short questions on length (in cm) and weight (in kg). Measuring eating behaviors in a reliable way is challenging, since dietary prescriptions differ per disease and individual patient [7, 8]. To avoid extensive questionnaires, we decided to measure adherence to a healthy diet perceived by the respondent, using two questions: “In the past week, how often have you kept a healthy diet?” with scores on a 5-point scale from “never” to “always”, and “In the past week, how well do you believe you have kept a healthy diet?” on a 1-10 rating scale from “very badly” to “very well”. For smoking, respondents can indicate whether they smoke, and if so, how many units per day. Last, for medication adherence, many validated questionnaires measure barriers and beliefs associated with adherence, whereas for the aim of screening and selecting patients that actually experience suboptimal adherence, it is more suitable to measure actual medication-taking behavior. Therefore, we found the Simplified Medication Adherence Questionnaire [SMAQ; 10] most adequate. This questionnaire consists of four dichotomous yes/no items (e.g. “Do you ever forget to take your medicine?”) and two items to quantify omissions (e.g. “Thinking about last week, how often have you not taken your medicine?”). We translated the questionnaire from Spanish to Dutch with a back translation approach, with help from bilingual researchers [11].

To detect an increased-risk profile, for psychological distress, the cut-off points of the original scales were used [3]: On the PHQ-9, cut-off points of 5, 10, 15, and 20 represent mild, moderate, moderately severe, and severe depressive symptom levels; On the GAD-7, cut-off points of 5, 10, and 15 represent mild, moderate, and severe anxiety symptom levels [3]. PHQ-9 and/or GAD-7 ≥ 5 thus were the cut-offs for psychological distress scores. Importantly, based the instruction manual of the scales and on previous experiences regarding suitability of web-based treatment for patients with severe psychological complaints [12], the research team decided that patients with severe distress scores (PHQ-9 ≥ 20 or GAD-7 ≥ 15) should be advised to contact their general practitioner for further evaluation and referral to specialized face-to-face mental health care. For HRQoL, the Hays norm-based scoring algorithm was applied [6], transforming raw RAND SF-36 scores into T-scores, and a standard deviation-derived cut-off score was used [13], by subtracting 0.5 SD from the norm mean [M=50±10; 6]. For the HRQoL subscales, scores ≤45 were thus used as cut-off points to determine an increased risk. For fatigue, the cut-off point of the original scale was used that indicates above-average fatigue, that is, SFQ scores ≥9 [5]. For most lifestyle behaviors, the cut-off points for suboptimal behaviors were based on the recommendations in lifestyle-related chronic diseases [7, 8], that is, < 150 minutes per week of moderate-to-vigorous intensity physical activity, a BMI ≥ 25, and/or tobacco smoking ≥ 1 unit per day. The research team determined the cut-off point to detect an unhealthy diet as follows: Respondents who perceive their adherence to a healthy diet in the past week as “never”, “seldom” or “half the time”, with a rating of ≤ 6 on the 1-10 scale. For medication adherence, the authors of the original SMAQ consider a patient to be non-adherent when at least one of the four dichotomous items was answered non-adherent, or > two doses missed over the past week, or >2 days of total non-medication during the past 3 months [10]. However, this was found a very strict cut-off, which could lead to an inclusion of patients who do not need support to improve their adherence. Therefore, it was decided that at least two items instead of one should indicate non-adherence.

***Part 2: Questionnaires for Intervention-Tailoring.*** Based on previous experiences in our research team regarding relevant information for intervention-tailoring [14], we selected specific areas of behavioral, psychological, social, and physical functioning: chronic condition self-management [Partners in Health Scale; 15], sleep quality [Medical Outcomes Study Sleep Scale; 16], illness cognitions [Illness Cognition Questionnaire; 17] and perceptions [Brief Illness Perception Questionnaire; 18], perceived stress [Perceived Stress Scale; 19], worrying [Penn State Worry Questionnaire; 20], optimism [Life Orientation Test–Revised; 21], neuroticism [NEO Personality Inventory–Revised Neuroticism; 22], and social support [Inventory for Social Reliance; 23]. Furthermore, we developed a short Personalized Priority and Progress Questionnaire (PPPQ) to measure patients’ personal priorities for improvement as well as actual subjective improvements over time in different areas of functioning (7 items) and lifestyle behaviors (5 items). This questionnaire is based on validated goal setting measurements [24-26]. In the items assessing priorities for functioning, respondents are asked to what degree they experienced limitations in different areas of functioning over the past 2 weeks, such as fatigue, anxiety, or daily activities, with response options from 1 “not at all” to 5 “very much”. In the items assessing priorities for lifestyle, respondents are asked to what degree they were able to keep a healthy lifestyle over the past two weeks, such as healthy eating and frequent physical activity, with response options from 1 “not at all” to 5 “very well”. In both scales, respondents are asked to prioritize 2 areas they would prefer to improve. At subsequent time points (e.g. three and six months later), respondents are asked to indicate any worsening or improvement per area over time on a 7-point scale from -3 to +3, on which 0 indicates neither worsening nor improvement. The development and validation of the PPPQ will be described in more detail in another manuscript by the research team (J.T., C.C., S.D., A.E., and H.M.), which is currently in preparation. Last, to measure self-efficacy for disease management, we translated the Self-Efficacy for Managing Chronic Disease 6-item Scale [27] and the Manage Disease in General Scale (5-item subscale from the Stanford Chronic Disease Self-Efficacy Scales) [28], hereafter called Stanford scales, from English to Dutch with a back translation approach, with help from a bilingual researcher [11]. These two scales contain three overlapping items. Depending on the patient population, the most appropriate scale could be used (e.g. the 6-item scale contains items about fatigue and pain, which may be less relevant for populations with largely asymptomatic diseases).

**References**

1. Kroenke, K.; Spitzer, R. L.; Williams, J. B. W. The PHQ-9 - Validity of a brief depression severity measure. *J Gen Intern Med* **2001,** *16*(9), 606-613, 10.1046/j.1525-1497.2001.016009606.x.

2. Spitzer, R. L.; Kroenke, K.; Williams, J. B. W.; Lowe, B. A brief measure for assessing generalized anxiety disorder - The GAD-7. *Arch Intern Med* **2006,** *166*(10), 1092-1097, 10.1001/archinte.166.10.1092.

3. Kroenke, K.; Spitzer, R. L.; Williams, J. B. W.; Lowe, B. The Patient Health Questionnaire somatic, anxiety, and depressive symptom scales: a systematic review. *Gen Hosp Psychiatry* **2010,** *32*(4), 345-359, 10.1016/j.genhosppsych.2010.03.006.

4. de Goeij, M. C. M.; Rotmans, J. I.; Eijgenraam, J. W.; Dekker, F. W.; Halbesma, N. Course of symptoms and health-related quality of life during specialized pre-dialysis care. *PLoS One* **2014,** *9*(4), e93069, 10.1371/journal.pone.0093069.

5. Alberts, M., Smets-Elshuis, E. M. A., Vercoulen, J. H. M. M., Garssen, B., & Bleijenberg, G. 'Verkorte vermoeidheidsvragenlijst': een praktisch hulpmiddel bij het scoren van vermoeidheid. *Nederlands Tijdschrift voor Geneeskunde* **1997,** *141*(31), 1526-1530.

6. Hays, R. D.; Sherbourne, C. D.; Mazel, R. M. The RAND 36-Item Health Survey 1.0. *Health Econ* **1993,** *2*(3), 217-227, 10.1002/hec.4730020305.

7. American Diabetes Association Lifestyle management: standards of medical care in diabetes-2019. *Diabetes Care* **2019,** *42*, S46-S60, 10.2337/dc19-S005.

8. KDIGO CKD Work Group KDIGO 2012 clinical practice guideline for the evaluation and management of chronic kidney disease. *Kidney Int* **2013,** *3*(1), 1-150, 10.1038/kisup.2012.72.

9. Wendel-Vos, G. C.; Schuit, A. J.; Saris, W. H.; Kromhout, D. Reproducibility and relative validity of the short questionnaire to assess health-enhancing physical activity. *J Clin Epidemiol* **2003,** *56*(12), 1163-1169.

10. Knobel, H.; Alonso, J.; Casado, J. L.; Collazos, J.; Gonzalez, J.; Ruiz, I.; Kindelan, J. M.; Carmona, A.; Juega, J.; Ocampo, A.; Group, G. S. Validation of a simplified medication adherence questionnaire in a large cohort of HIV-infected patients: the GEEMA Study. *Aids* **2002,** *16*(4), 605-613, 10.1097/00002030-200203080-00012.

11. Weeks, A.; Swerissen, H.; Belfrage, J. Issues, challenges, and solutions in translating study instruments. *Eval Rev* **2007,** *31*(2), 153-165, 10.1177/0193841x06294184.

12. Ciere, Y.; van der Vaart, R.; van der Meulen-De Jong, A. E.; Maljaars, P. W. J.; van Buul, A. R.; Koopmans, J. G.; Snoeck-Stroband, J. B.; Chavannes, N. H.; Sont, J. K.; Evers, A. W. M. Implementation of an eHealth self-management care path for chronic somatic conditions. *Clinical eHealth* **2019,** *2*, 3-11, 10.1016/j.ceh.2019.04.001.

13. Ward, R.; Mackey, D., Norms & Rating scales. In *Inquiry and measurement in kinesiology*, 2nd ed.; Biomedical Physiology & Kinesiology, Simon Fraser University: British Columbia, 2013.

14. Evers, A. W. M.; Gieler, U.; Hasenbring, M. I.; van Middendorp, H. Incorporating biopsychosocial characteristics into personalized healthcare: a clinical approach. *Psychother Psychosom* **2014,** *83*(3), 148-157, 10.1159/000358309.

15. Petkov, J.; Harvey, P.; Battersby, M. The internal consistency and construct validity of the partners in health scale: validation of a patient rated chronic condition self-management measure. *Qual Life Res* **2010,** *19*(7), 1079-1085, 10.1007/s11136-010-9661-1.

16. Hays, R. D., & Stewart, A. L., Sleep measures. In *Measuring Functioning and Well-being: The Medical Outcomes Study Approach.*, Stewart, A. L., & Ware, J. E., Ed. Duke University Press: Durham, NC, 1992; pp 235-259.

17. Evers, A. W. M.; Kraaimaat, F. W.; van Lankveld, W.; Jongen, P. J.; Jacobs, J. W.; Bijlsma, J. W. Beyond unfavorable thinking: the illness cognition questionnaire for chronic diseases. *J Consult Clin Psychol* **2001,** *69*(6), 1026-36, 10.1037//0022-006X.69.6.1026.

18. Broadbent, E.; Petrie, K. J.; Main, J.; Weinman, J. The Brief Illness Perception Questionnaire. *J Psychosom Res* **2006,** *60*(6), 631-637, 10.1016/j.jpsychores.2005.10.020.

19. Cohen, S.; Kamarck, T.; Mermelstein, R. A global measure of perceived stress. *J Health Soc Behav* **1983,** *24*(4), 385-396, 10.2307/2136404.

20. Meyer, T. J.; Miller, M. L.; Metzger, R. L.; Borkovec, T. D. Development and validation of the Penn State Worry Questionnaire. *Behav Res Ther* **1990,** *28*(6), 487-495, 10.1016/0005-7967(90)90135-6.

21. Scheier, M. F.; Carver, C. S.; Bridges, M. W. Distinguishing optimism from neuroticism (and trait anxiety, self-mastery, and self-esteem): a reevaluation of the Life Orientation Test. *J Pers Soc Psychol* **1994,** *67*(6), 1063-1078.

22. Costa, P. T.; McCrae, R. R., *Revised NEO Personality Inventory (NEO-PI-R) and the Five Factor Inventory (NEO-FFI): Professional Manual*. Psychological Assessment Resources, Inc.: Odessa, FL, 1992.

23. Dam-Baggen, R.; Kraaimaat, F. W. De Inventarisatielijst Sociale Betrokkenheid (ISB): een zelfbeoordelingslijst om sociale steun te meten (Inventory for social reliance (ISR): a self-report inventory for the measurement of social support). *Gedragstherapie* **1992,** *25*, 27-46.

24. Little, B. R. Personal Projects - a rationale and method for investigation. *Environ Behav* **1983,** *15*(3), 273-309, 10.1177/0013916583153002.

25. Melville, L. L.; Baltic, T. A.; Bettcher, T. W.; Nelson, D. L. Patients' perspectives on the self-identified goals assessment. *Am J Occup Ther* **2002,** *56*(6), 650-659.

26. Tugwell, P.; Bombardier, C.; Buchanan, W. W.; Goldsmith, C. H.; Grace, E.; Hanna, B. The Mactar Patient Preference Disability Questionnaire - an individualized functional priority approach for assessing improvement in physical disability in clinical trials in rheumatoid arthritis. *J Rheumatol* **1987,** *14*(3), 446-451.

27. Lorig, K. R.; Sobel, D. S.; Ritter, P. L.; Laurent, D.; Hobbs, M. Effect of a self-management program on patients with chronic disease. *Eff Clin Pract* **2001,** *4*(6), 256-262.

28. Lorig, K.; Steward, A.; Ritter, P.; González, V.; Laurent, D.; Lynch, J., *Outcome measures for health education and other health care interventions*. Sage Publications: Thousand Oaks CA, 1996.
