# Supplementary material for: Detecting and Treating Psychosocial and Lifestyle-Related Difficulties in Chronic Disease: Development and Treatment Protocol of the E-GOAL eHealth Care Pathway
Source: Int J Environ Res Public Health. 2021 Mar 23;18(6):3292. doi: 10.3390/ijerph18063292 (PMC8005221; doi:10.3390/ijerph18063292)
Supplement: Supplementary file 1 [file ijerph-18-03292-s001.zip › 20210322_ijerph-1139819_S2_proofreading.docx]

**Supplementary File 2**

**Initial prototypes of personal profile charts and monitor charts**


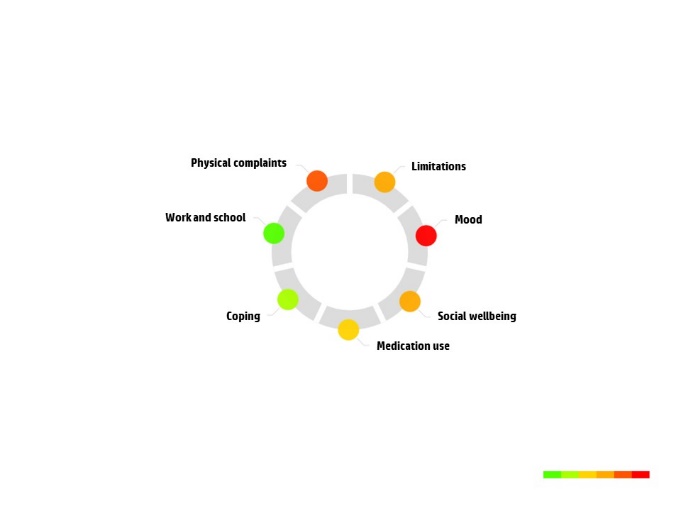

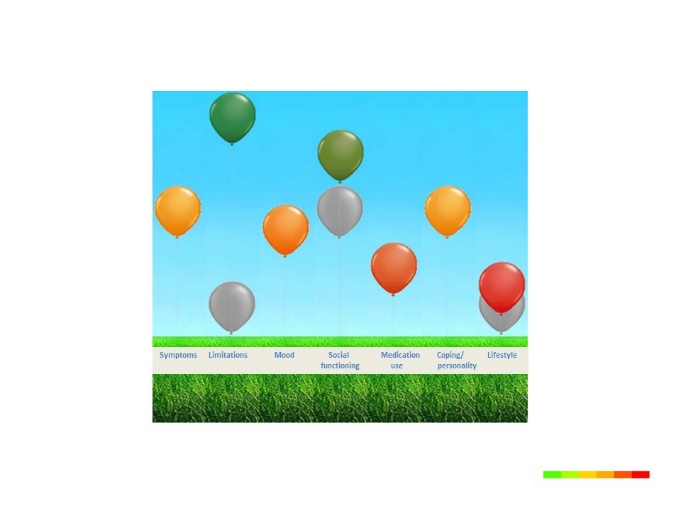

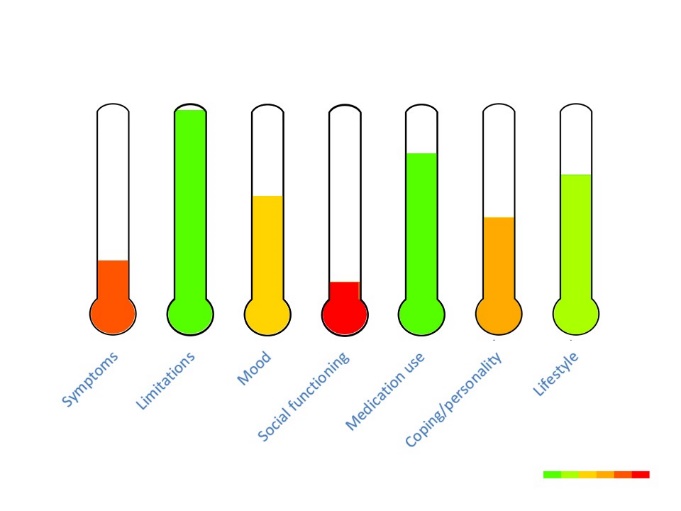


**(c)**

**(a)A**

**(b)**

**Figure B1.** Prototypes of profile charts: (**a**) wheel, (**b**) balloons, and (**c**) thermometers. Balloon image is adapted with permission from A. H. M. Slok et al., Effectiveness of the Assessment of Burden of COPD (ABC) tool on health-related quality of life in patients with COPD: a cluster randomised controlled trial in primary and hospital care; published by NPJ Primary Care Respiratory Medicine, 2014 [1].


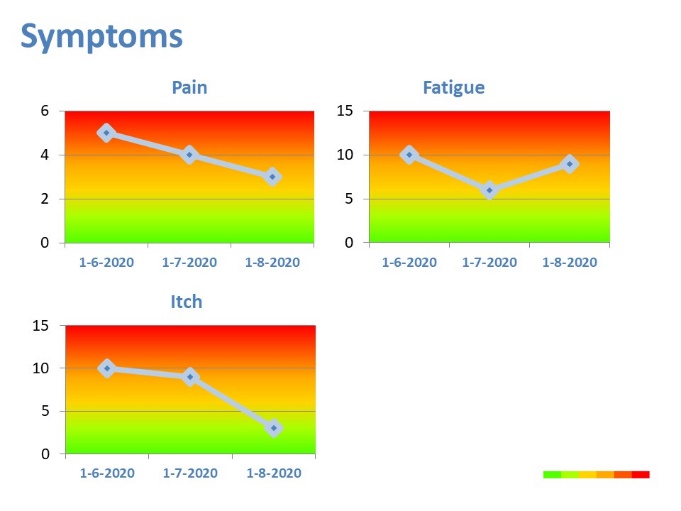

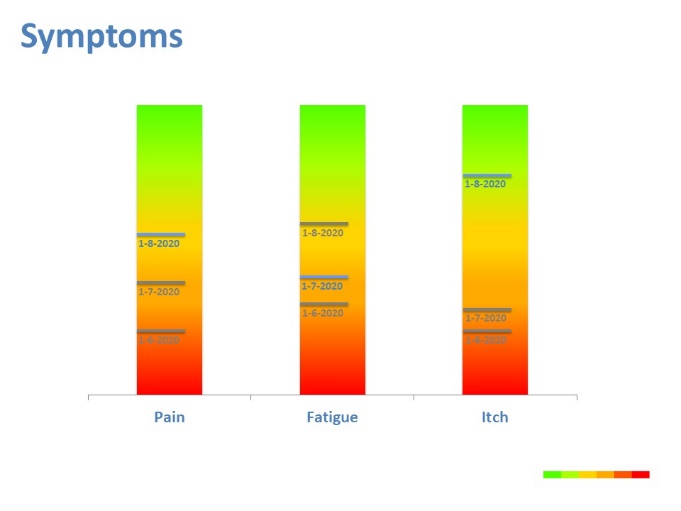


**(b)**

**(a)**

**Figure B2.** Prototypes of monitor charts: (**a**) line chart and (**b**) bar chart.

**References**

1. Slok, A. H.; in 't Veen, J. C.; Chavannes, N. H.; van der Molen, T.; Rutten-van Molken, M. P.; Kerstjens, H. A.; Salome, P. L.; Holverda, S.; Dekhuijzen, P. N.; Schuiten, D.; Asijee, G. M.; van Schayck, O. C. Development of the Assessment of Burden of COPD tool: an integrated tool to measure the burden of COPD. *NPJ Prim Care Respir Med* **2014,** *24*, 14021, 10.1038/npjpcrm.2014.21.
